# Supplementary material for: Targeting of the Mitochondrial TET1 Protein by Pyrrolo[3,2-b]pyrrole Chelators
Source: Int J Mol Sci. 2022 Sep 16;23(18):10850. doi: 10.3390/ijms231810850 (PMC9505425; doi:10.3390/ijms231810850)
Supplement: Supplementary file 1 [file ijms-23-10850-s001.zip › ijms-1854344-supplementary.pdf]

Targeting of the Mitochondrial TET1 Protein by Pyrrolo[3,2-*b*]pyrrole Chelators

Veronika Antonyová [1,2], Ameneh Tatar [1,3], Tereza Brogyányi [1,4], Zdeněk Kejík [1,2,3], Robert Kaplánek [1,2,3], Fréderic Vellieux [2], Nikita Abramenko [1,2], Alla Sinica [2,3], Jan Hajduch [2,3], Petr Novotný [1,2], Bettie Sue Masters [5], Pavel Martásek [1,*] and Milan Jakubek [1,2,3,*]

[1] Department of Paediatrics and Inherited Metabolic Disorders, First Faculty of Medicine, Charles University and General University Hospital in Prague, Ke Karlovu 455/2, 128 08 Prague, Czech Republic
[2] BIOCEV, First Faculty of Medicine, Charles University, 252 20 Vestec, Czech Republic
[3] Department of Analytical Chemistry, Faculty of Chemical Engineering, University of Chemistry and Technology, 166 28 Prague, Czech Republic
[4] Institute of Pathological Physiology, First Faculty of Medicine, Charles University, 128 53 Prague, Czech Republic
[5] Duke University Medical Center, Department of Biochemistry, Durham, NC 27707, USA
* Correspondence: pavel.martasek@lf1.cuni.cz (P.M.); milan.jakubek@lf1.cuni.cz (M.J.)

Supplementary Materials

Contents

Figure S1. NMR spectra of Compound **1** in DMSO-d_6.
Figure S2. NMR spectra of Compound **2** in DMSO-d_6.
Figure S3. NMR spectra of Compound **3** in DMSO-d_6.
Figure S4. NMR spectra of Compound **4** in DMSO-d_6.
Figure S5. NMR spectra of Compound **5** in DMSO-d_6.
Figure S6. NMR spectra of Compound **6** in DMSO-d_6.
Figure S7. NMR spectra of Compound **7** in DMSO-d_6.
Figure S8. HRMS spectra of Compound **1**.
Figure S9. HRMS spectra of Compound **2**.
Figure S10. HRMS spectra of Compound **3**.
Figure S11. HRMS spectra of Compound **4**.
Figure S12. HRMS spectra of Compound **5**.
Figure S13. HRMS spectra of Compound **6**.
Figure S14. HRMS spectra of Compound **7**.
Figure S15. Close up view of the TET1 active site showing docked compound **2** in a sphere representation.
Figure S16. Close up view of the TET1 active site showing docked compound **3** in a sphere representation.
Figure S17. Close up view of the TET1 active site showing docked compound **4** in a sphere representation.
Figure S18. Close up view of the TET1 active site showing docked compound **5** in a sphere representation.
Figure S19. Close up view of the TET1 active site showing docked compound **6** in a sphere representation.
Figure S20. Close up view of the TET1 active site showing docked compound **7** in a sphere representation.
Table S1. Residual TET1 protein activity on the concentration of compound **1.**
Table S2. Viability of Caov-3 cells in the presence of compound **1**.
Table S3. Viability of Caov-3 cells in the presence of compound **2**.
Table S4. Viability of Caov-3 cells in the presence of compound **3**.

Table S5. Viability of Caov-3 cells in the presence of compound **4**.
Table S6. Viability of Caov-3 cells in the presence of compound **5**.
Table S7. Viability of Caov-3 cells in the presence of compound **6**.
Table S8. Viability of H-1299 cells in the presence of compound **1**.
Table S9. Viability of H-1299 cells in the presence of compound **2**.
Table S10. Viability of H-1299 cells in the presence of compound **3**.
Table S11. Viability of H-1299 cells in the presence of compound **4**.
Table S12. Viability of H-1299 cells in the presence of compound **5**.
Table S13. Viability of H-1299 cells in the presence of compound **6**.
Table S14. Viability of BT-20 cells in the presence of compound **1**.
Table S15. Viability of BT-20 cells in the presence of compound **2**.
Table S16. Viability of BT-20 cells in the presence of compound **3**.
Table S17. Viability of BT-20 cells in the presence of compound **4**.
Table S18. Viability of BT-20 cells in the presence of compound **5**.
Table S19. Viability of BT-20 cells in the presence of compound **6**.
Table S20. Viability of U-118 MG cells in the presence of compound **1**.
Table S21. Viability of U-118 MG cells in the presence of compound **2**.
Table S22. Viability of U-118 MG cells in the presence of compound **3**.
Table S23. Viability of U-118 MG cells in the presence of compound **4**.
Table S24. Viability of U-118 MG cells in the presence of compound **5**.
Table S25. Viability of U-118 MG cells in the presence of compound **6**.
Table S26. Viability of HF-P4 cells in the presence of compound **1**.
Table S27. Viability of HF-P4 cells in the presence of compound **2**.
Table S28. Viability of HF-P4 cells in the presence of compound **3**.
Table S29. Viability of HF-P4 cells in the presence of compound **4**.
Table S30. Viability of HF-P4 cells in the presence of compound **5**.
Table S31. Viability of HF-P4 cells in the presence of compound **6**.

NMR spectra of Compound 1–7 in DMSO-d6

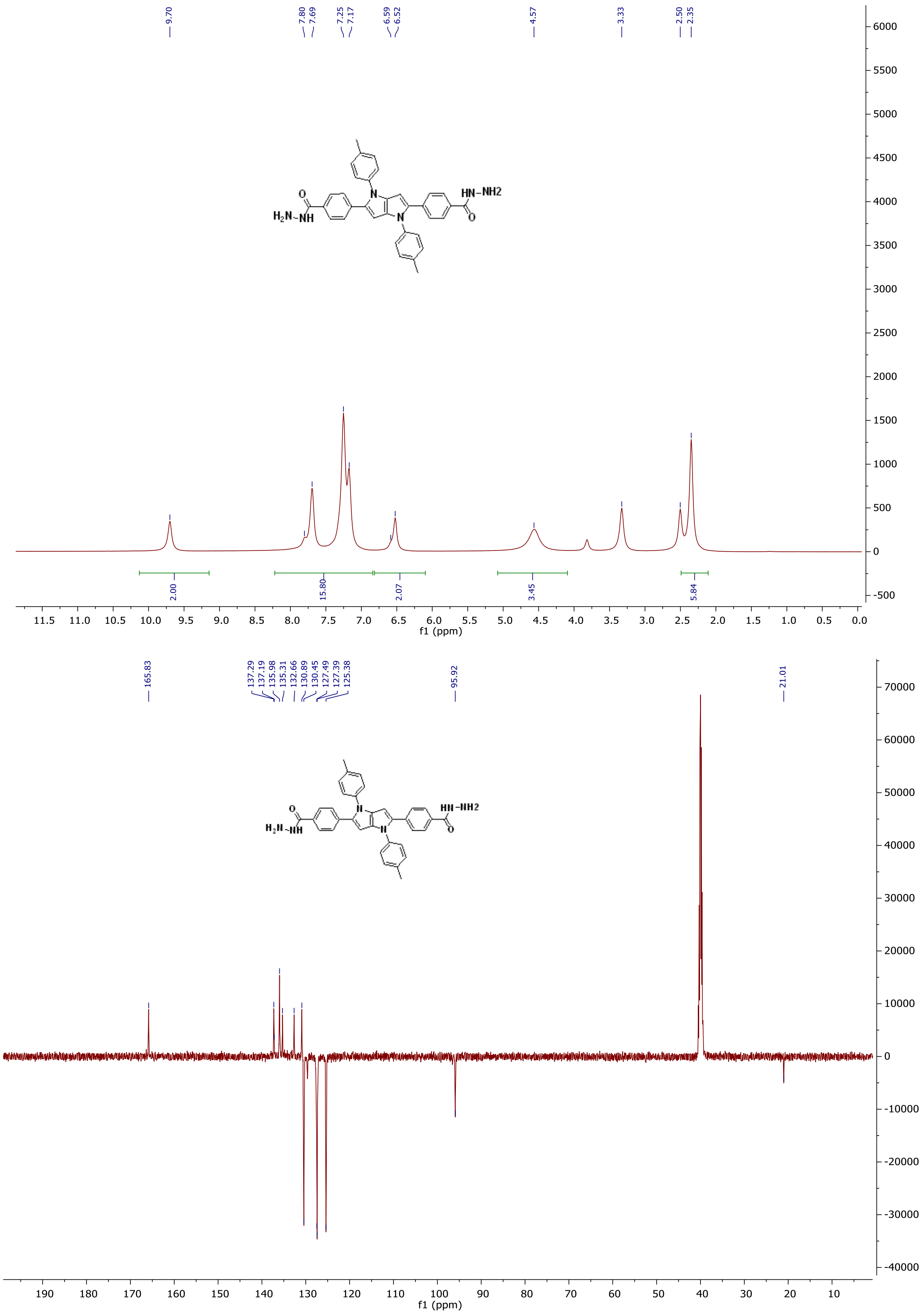

Figure S1. ^{1}HNMR and ^{13}CNMR spectra of Compound **1** in DMSO-d6.

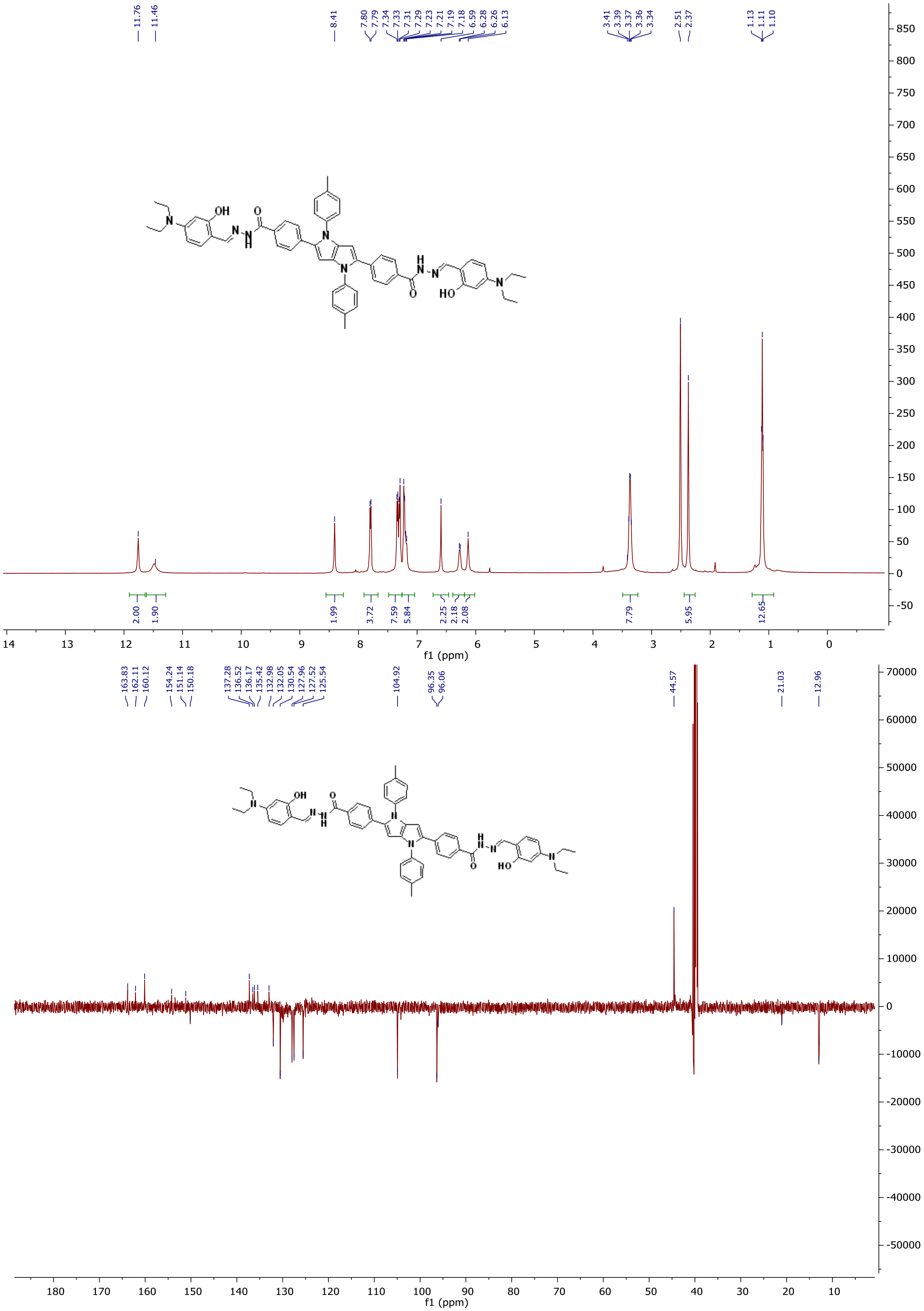

Figure S2. ^{1}HNMR and ^{13}CNMR spectra of Compound **2** in DMSO-d6.

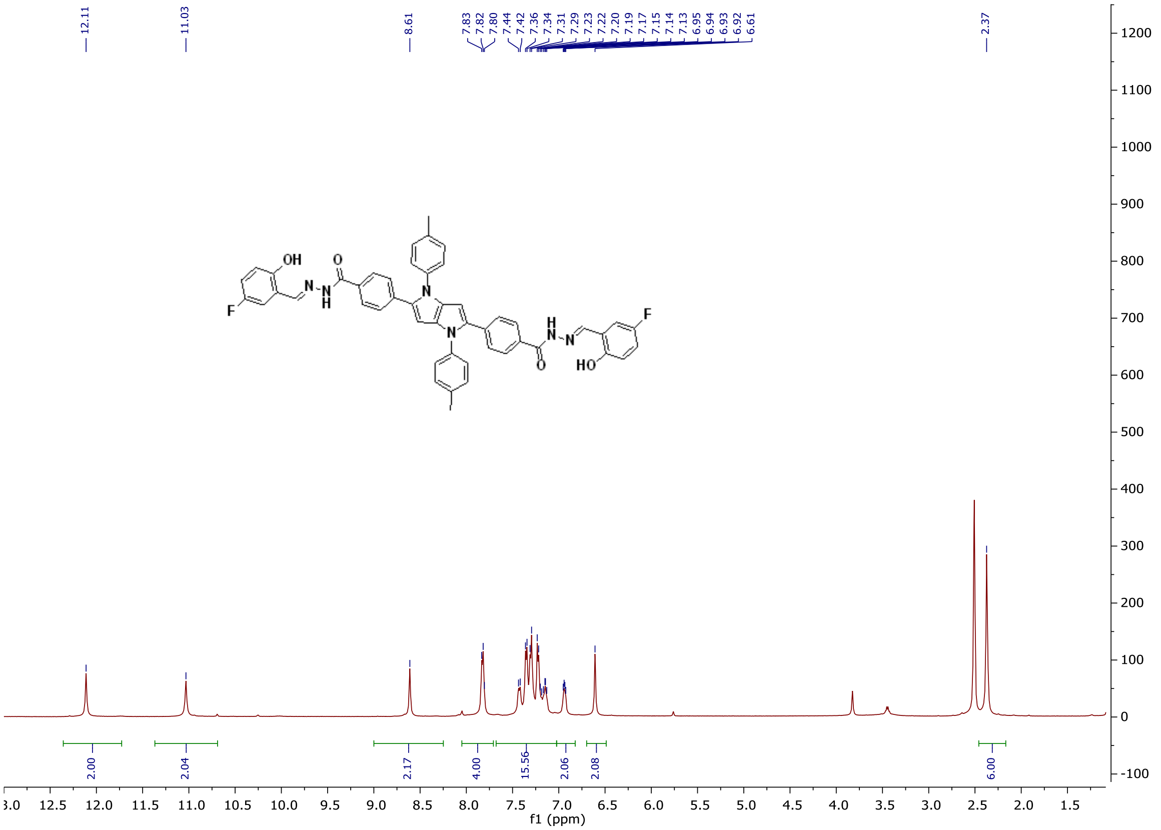

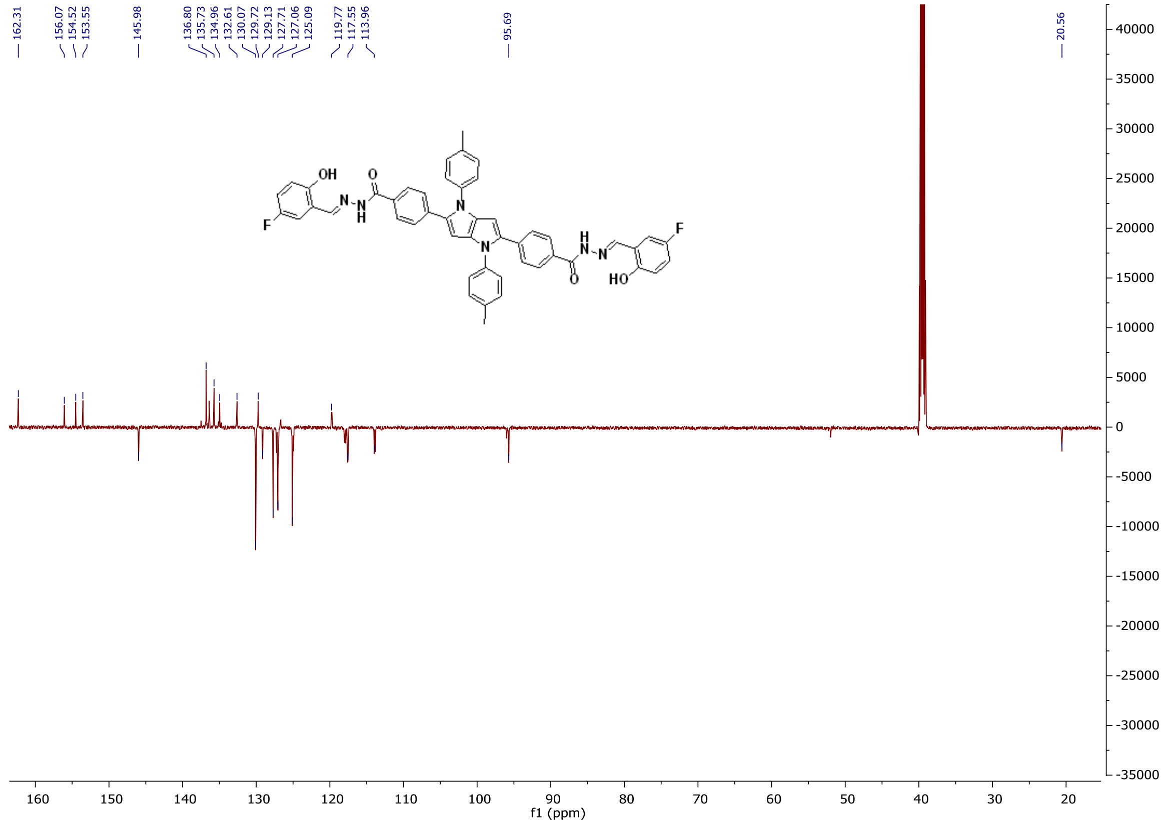

Figure S3. ^{1}HNMR and ^{13}CNMR spectra of Compound **3** in DMSO-d_6.

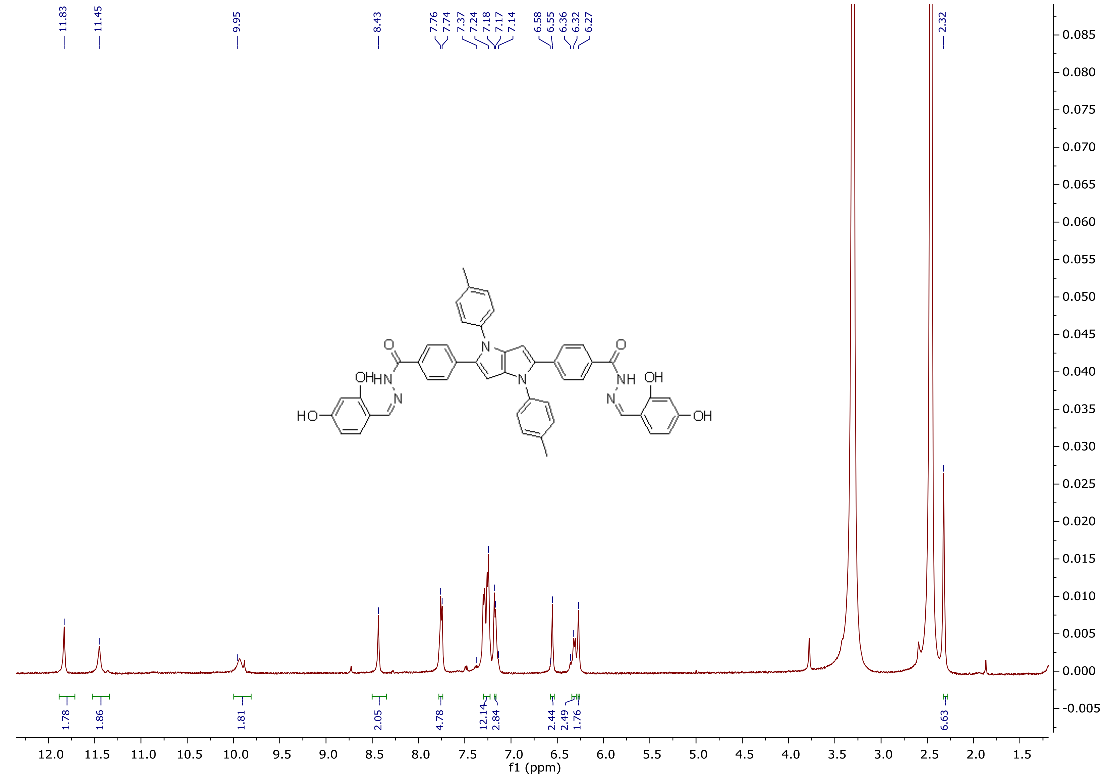

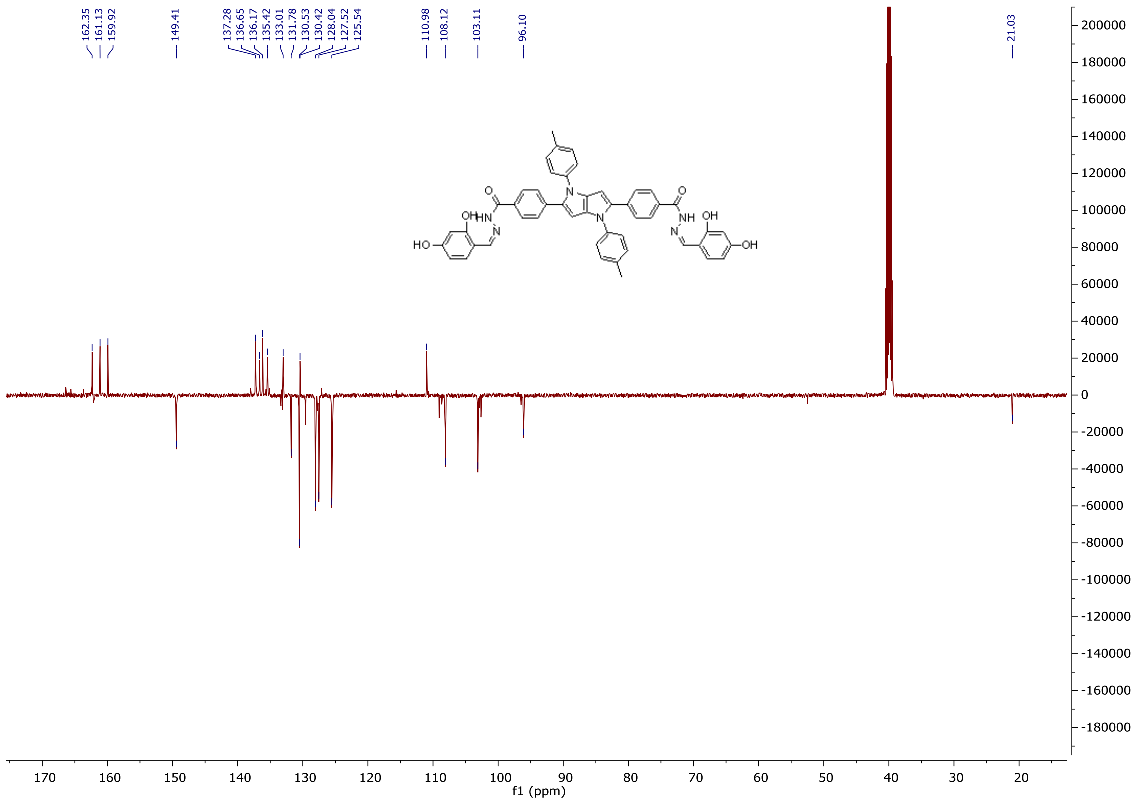

Figure S4. ^{1}HNMR and ^{13}CNMR spectra of Compound **4** in DMSO-d_6.

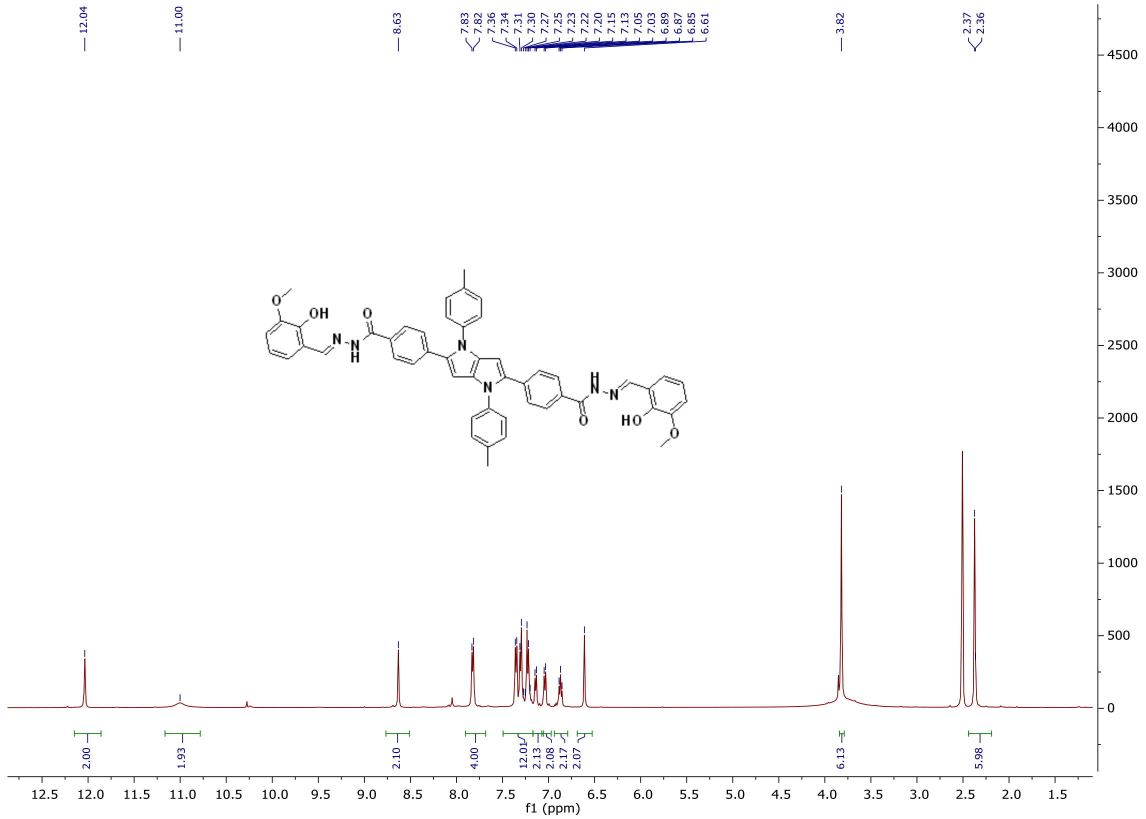

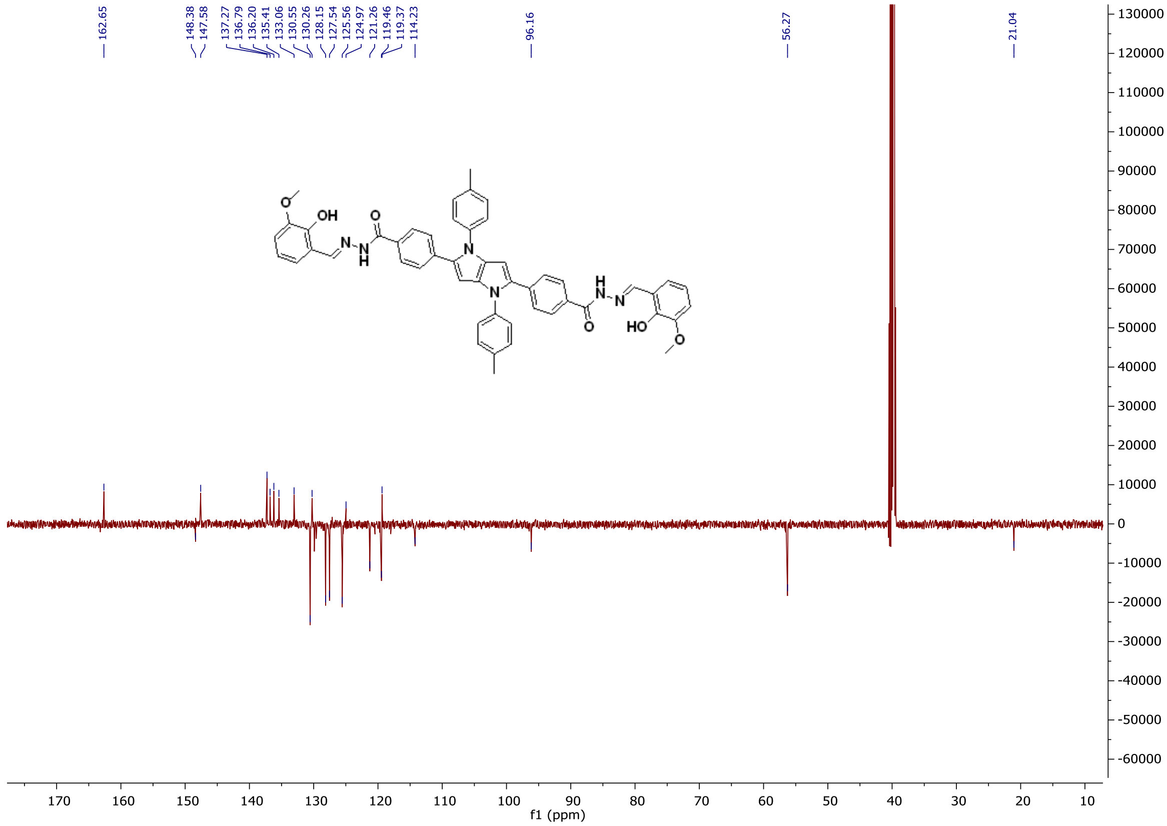

Figure S5. ^{1}HNMR and ^{13}CNMR spectra of Compound **5** in DMSO-**d6.**

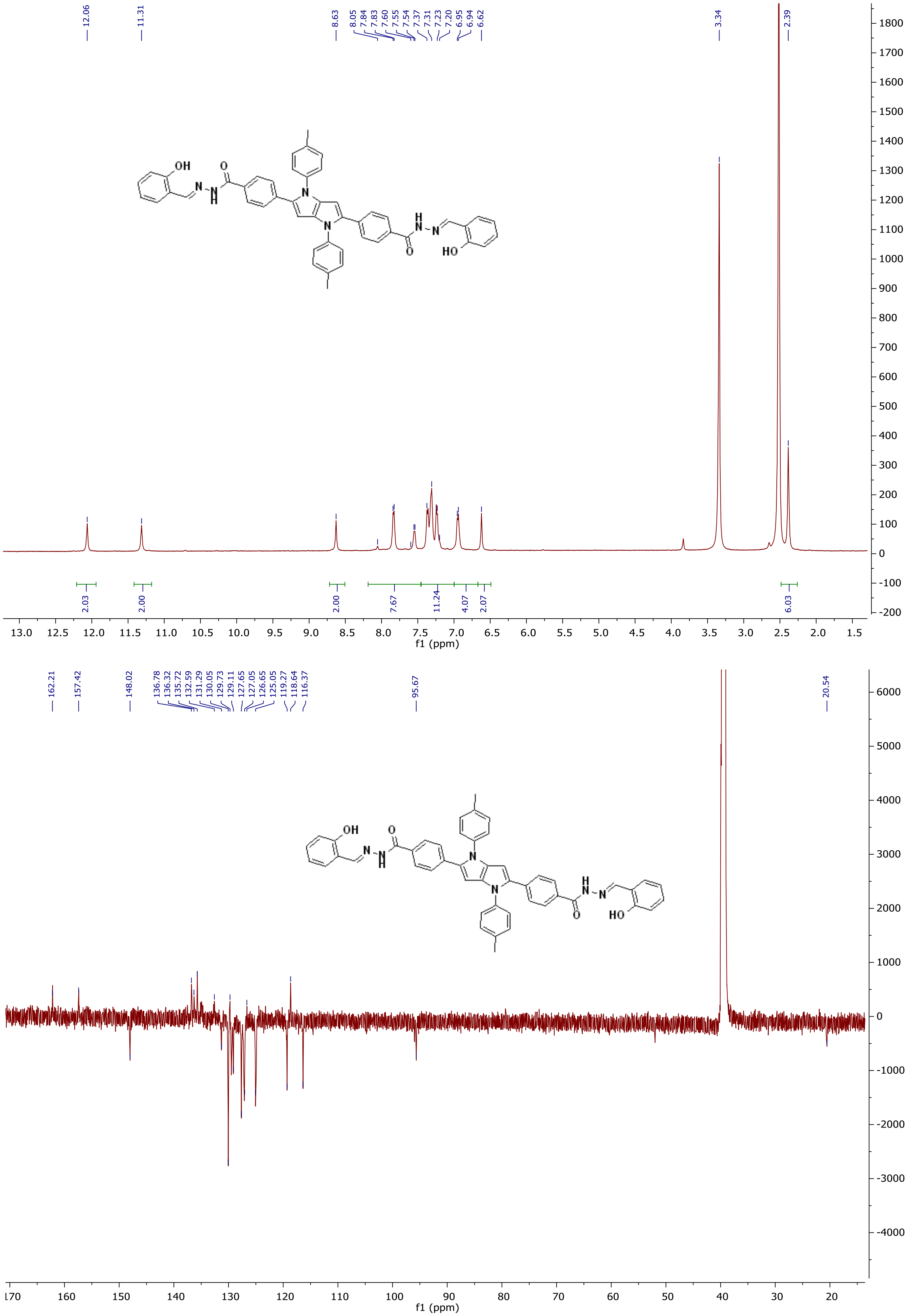

Figure S6. ^{1}HNMR and ^{13}CNMR spectra of Compound **6** in DMSO-d$_6$.

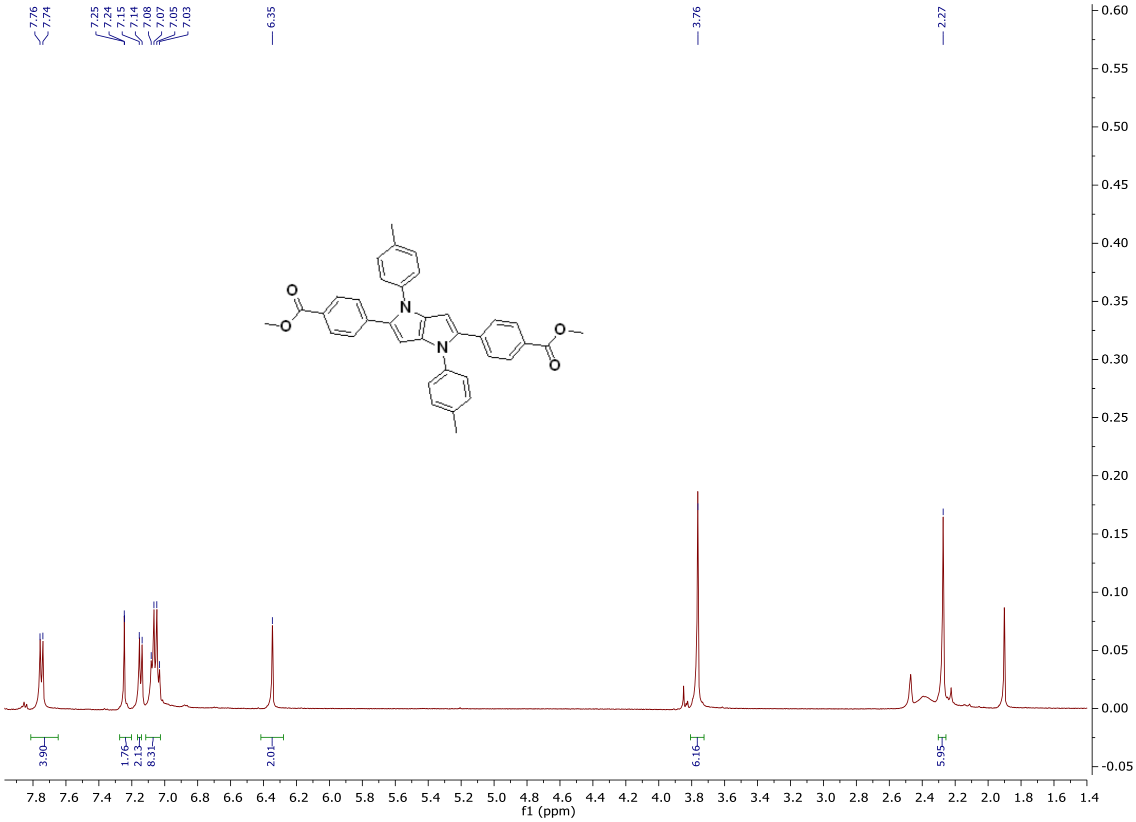

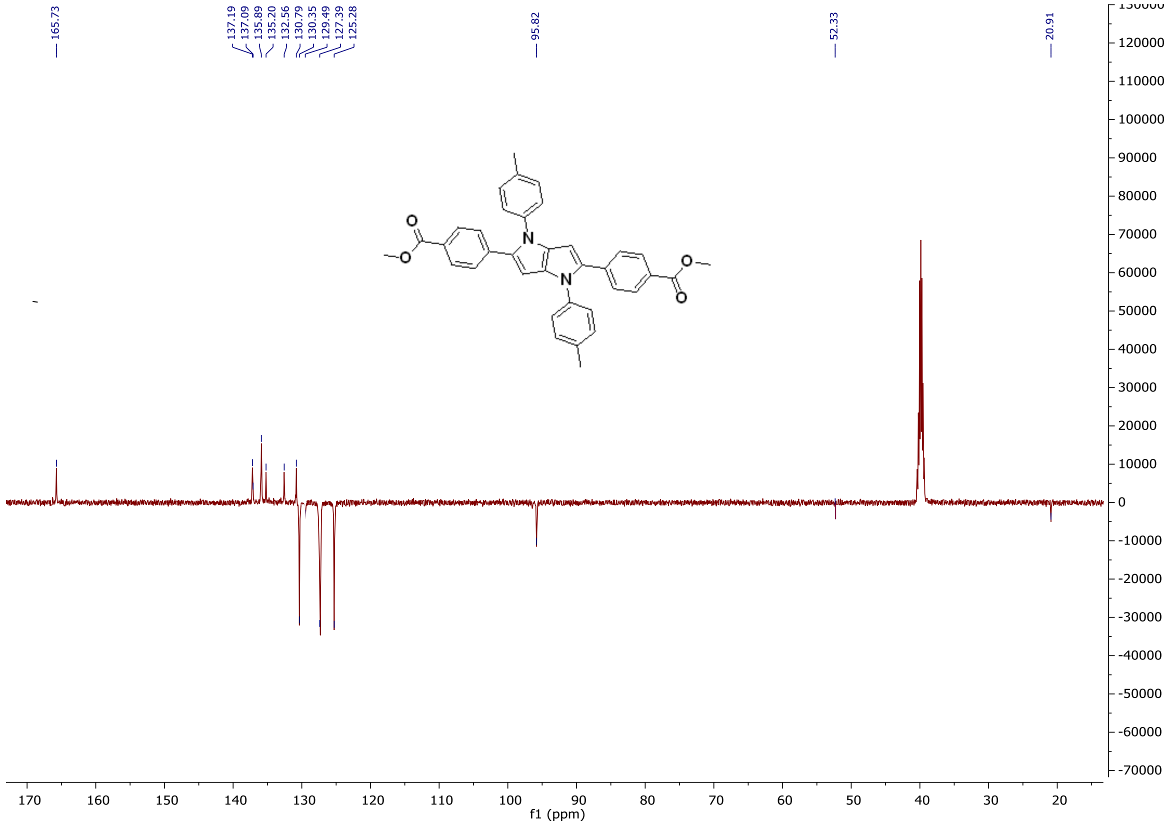

Figure S7. ^{1}HNMR and ^{13}CNMR spectra of Compound **7** in DMSO-d_6.

HRMS spectra of compound 1–7

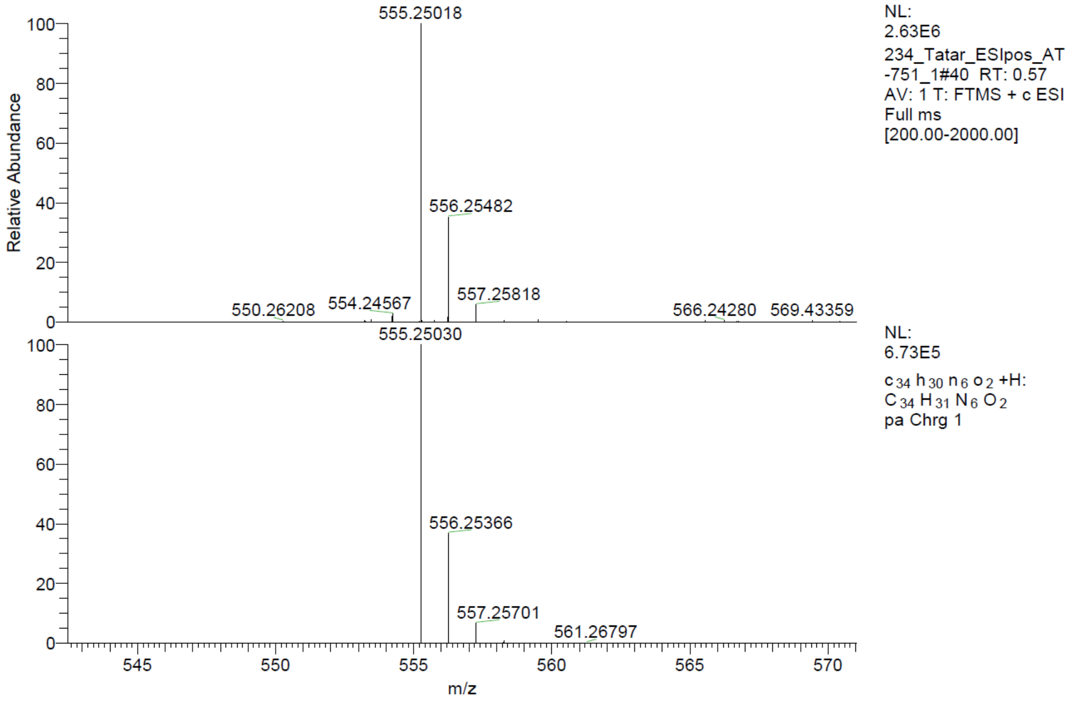

Figure S8. MS spectra of compound **1**

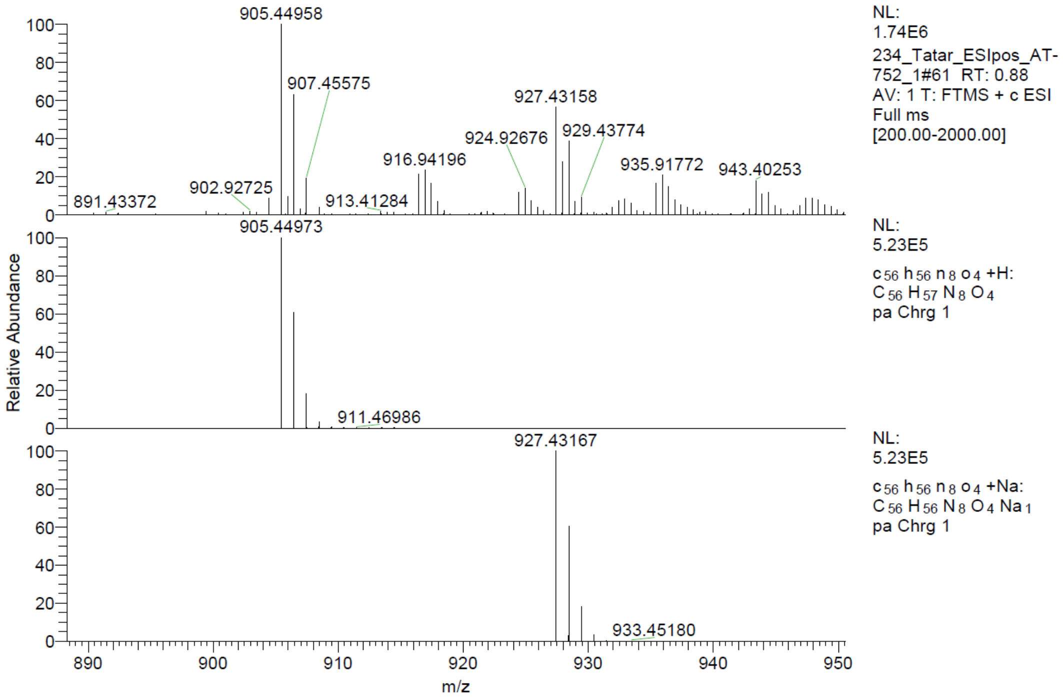

Figure S9. MS spectra of compound **2.**

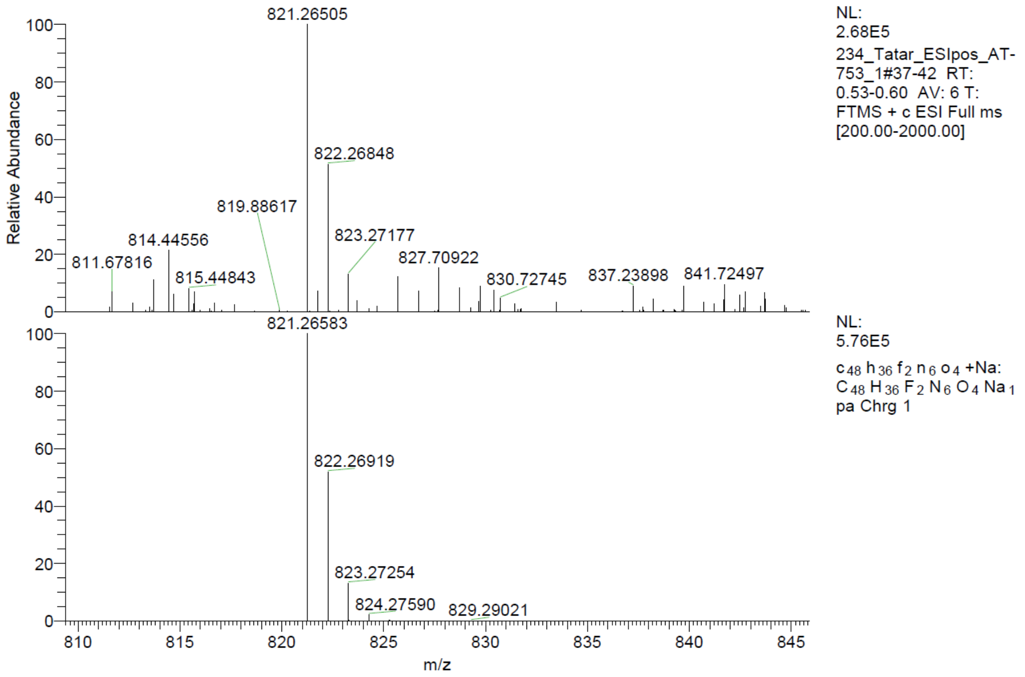

Figure S10. MS spectra of compound **3.**

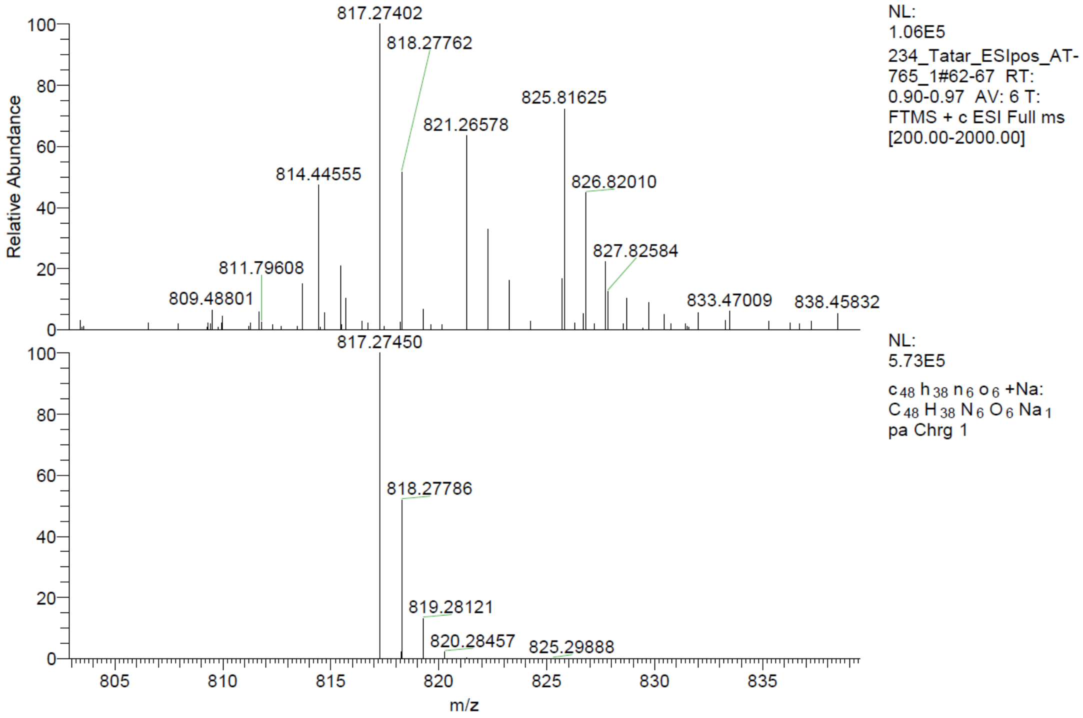

Figure S11. MS spectra of compound **4.**

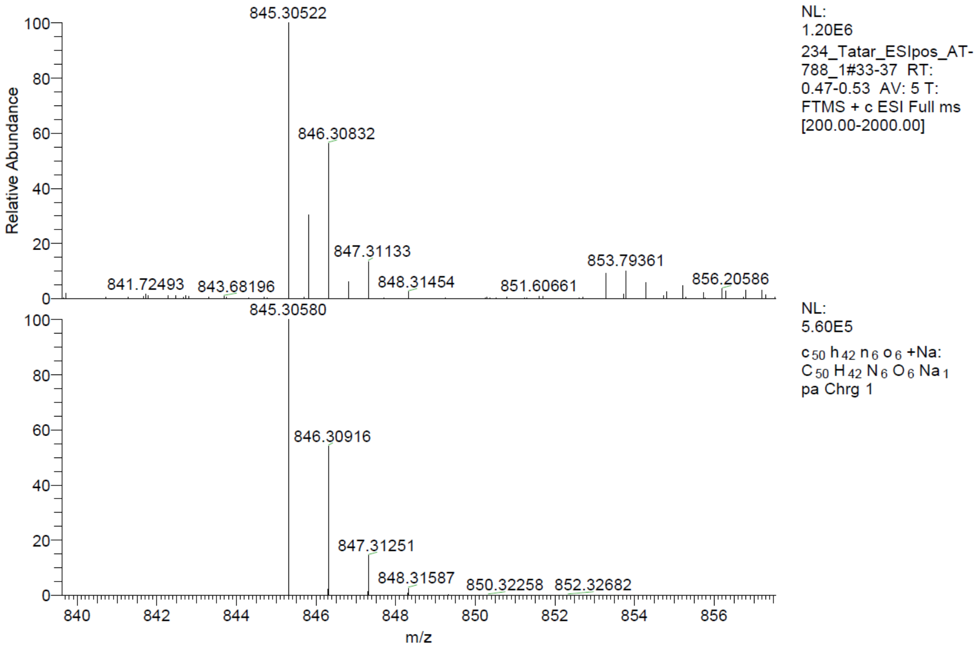

Figure S12. MS spectra of compound **5.**

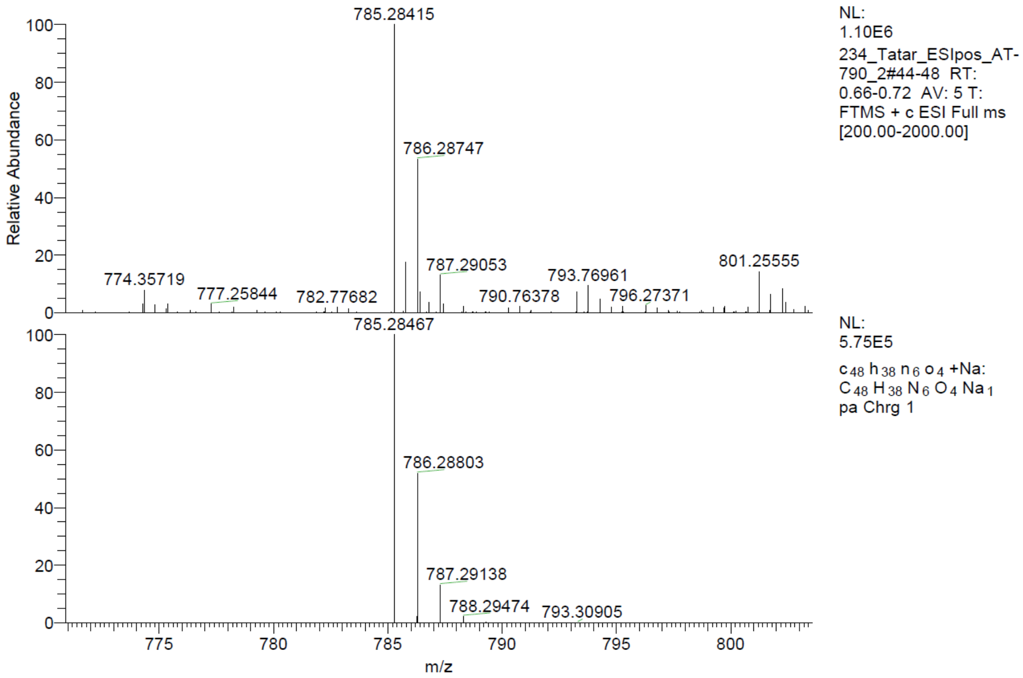

Figure S13. MS spectra of compound **6.**

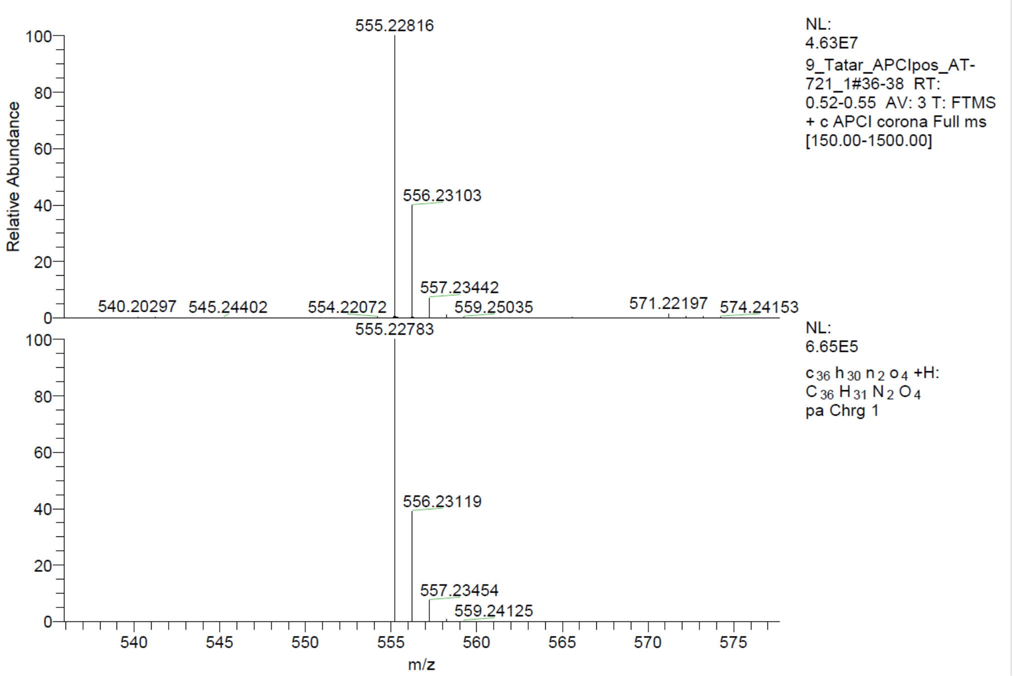

Figure S14. MS spectra of compound **7.**

Docking compounds 2–6 to the TET1 protein

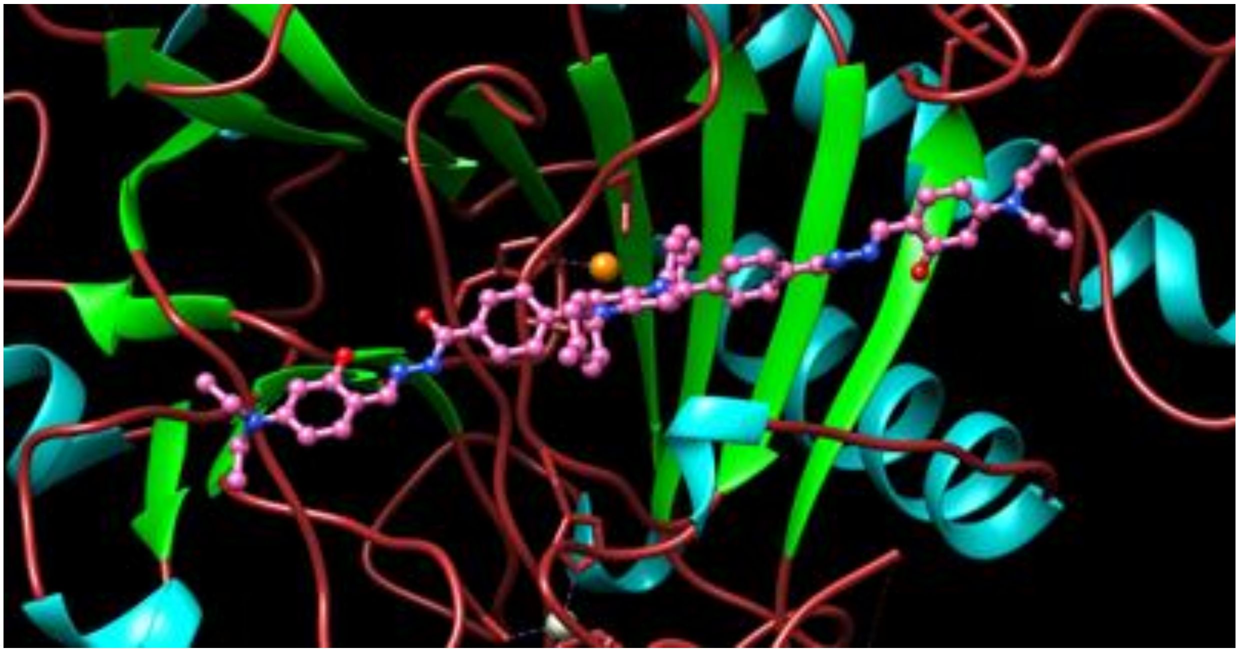

Figure S15. Close up view of the TET1 active site showing docked compound **2** in a sphere representation. The Fe(II) ion is displayed as a crossed orange sphere, the protein residues involved in contacts with compound **2** (pink sticks) are shown as sticks. The hydrogen bonds and carbon hydrogen bond are indicated by purple and yellow dotted lines, respectively.

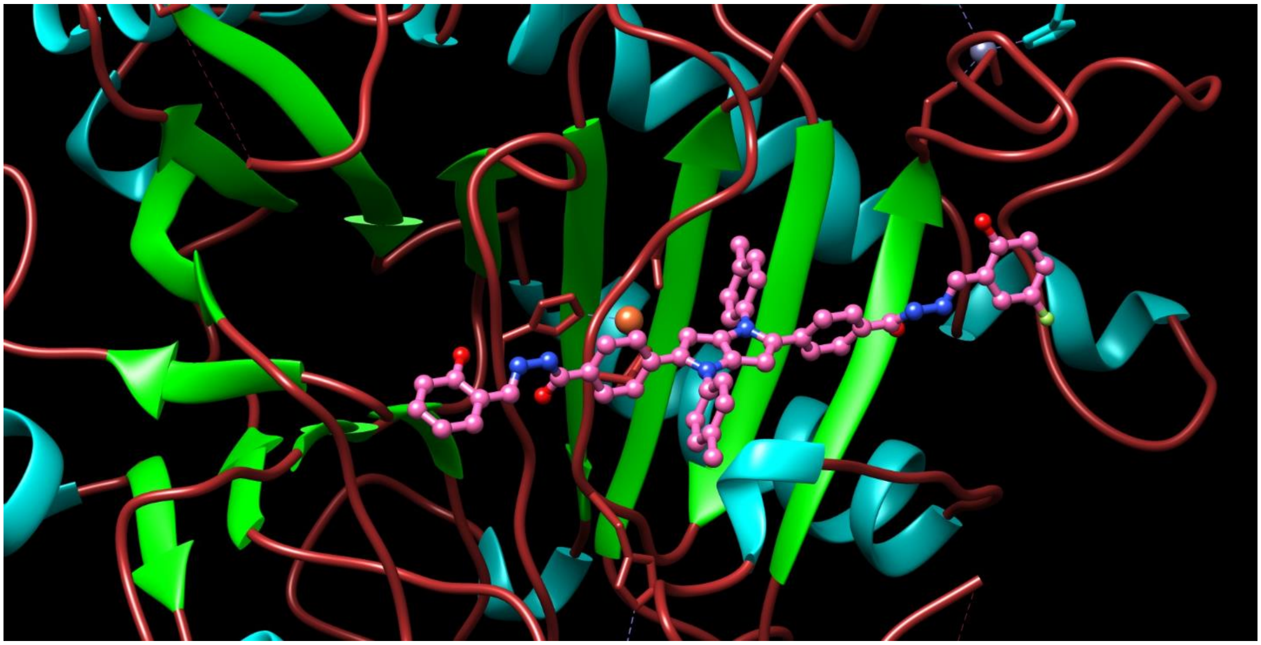

Figure S16. Close up view of the TET1 active site showing docked compound **3** in a sphere representation. The Fe(II) ion is displayed as a crossed orange sphere, the protein residues involved in contacts with compound **3** (pink sticks) are shown as sticks. The hydrogen bonds and carbon hydrogen bond are indicated by purple and yellow dotted lines, respectively.

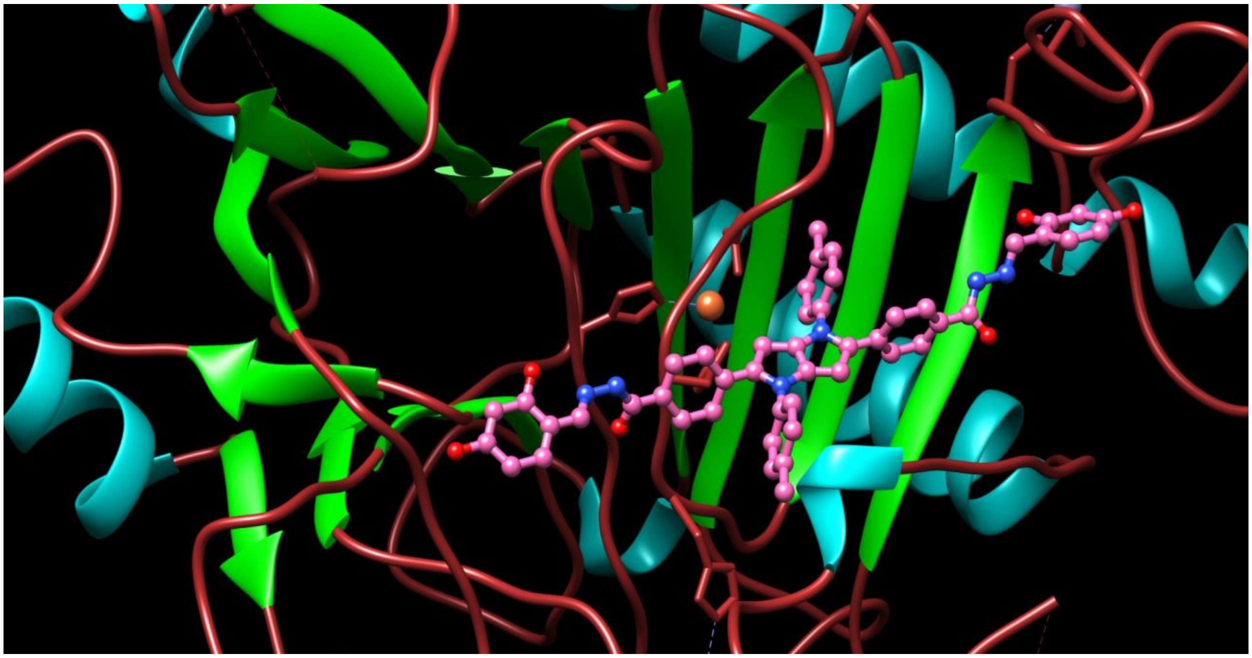

Figure S17. Close up view of the TET1 active site showing docked compound **4** in a sphere representation. The Fe(II) ion is displayed as a crossed orange sphere, the protein residues involved in contacts with compound **4** (pink sticks) are shown as sticks. The hydrogen bonds and carbon hydrogen bond are indicated by purple and yellow dotted lines, respectively.

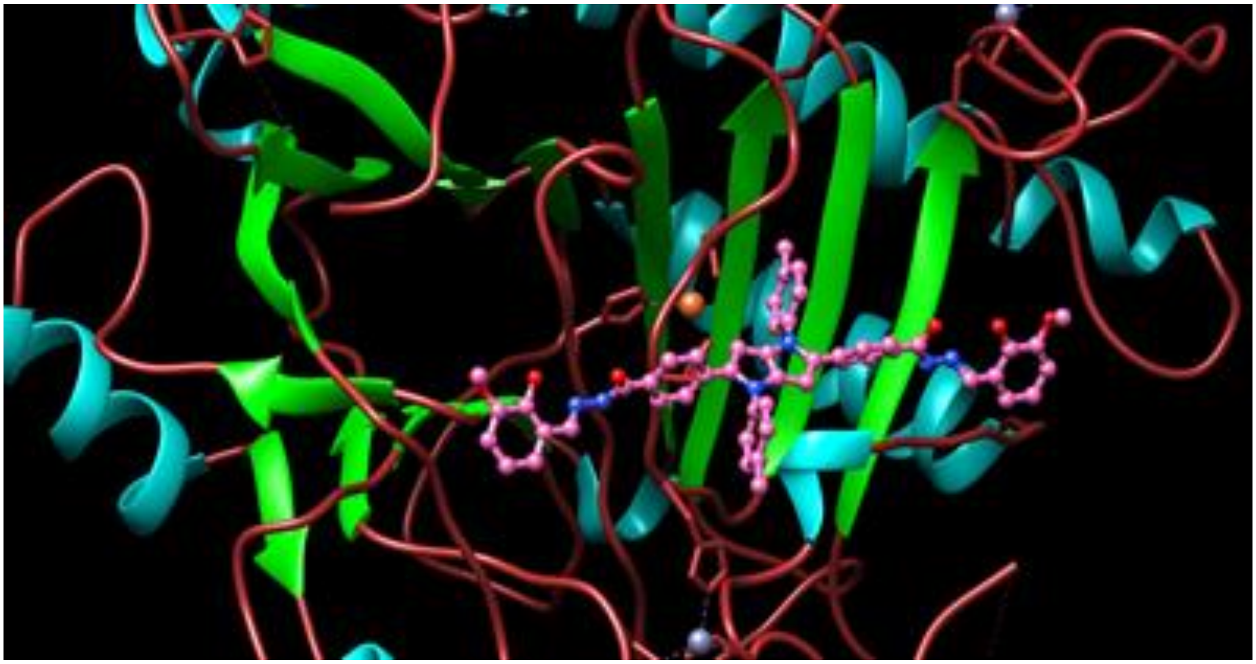

Figure S18. Close up view of the TET1 active site showing docked compound **5** in a sphere representation. The Fe(II) ion is displayed as a crossed orange sphere, the protein residues involved in contacts with compound **5** (pink sticks) are shown as sticks. The hydrogen bonds and carbon hydrogen bond are indicated by purple and yellow dotted lines, respectively.

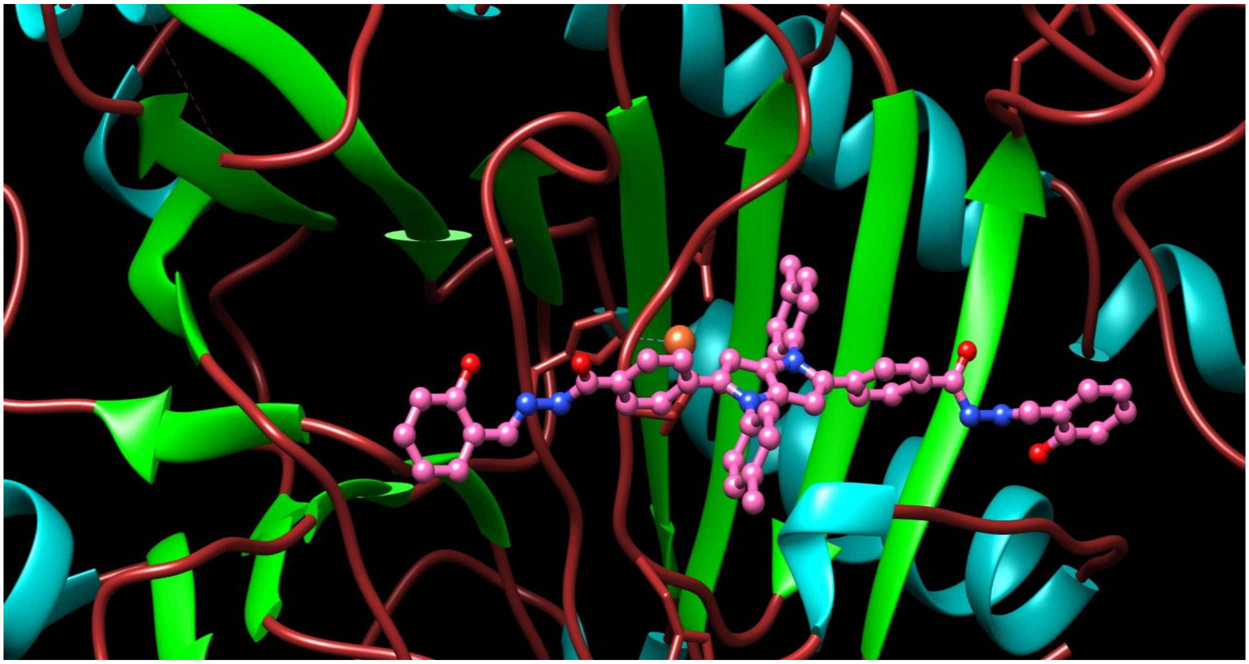

Figure S19. Close up view of the TET1 active site showing docked compound **6** in a sphere representation. The Fe(II) ion is displayed as a crossed orange sphere, the protein residues involved in contacts with compound **6** (pink sticks) are shown as sticks. The hydrogen bonds and carbon hydrogen bond are indicated by purple and yellow dotted lines, respectively.

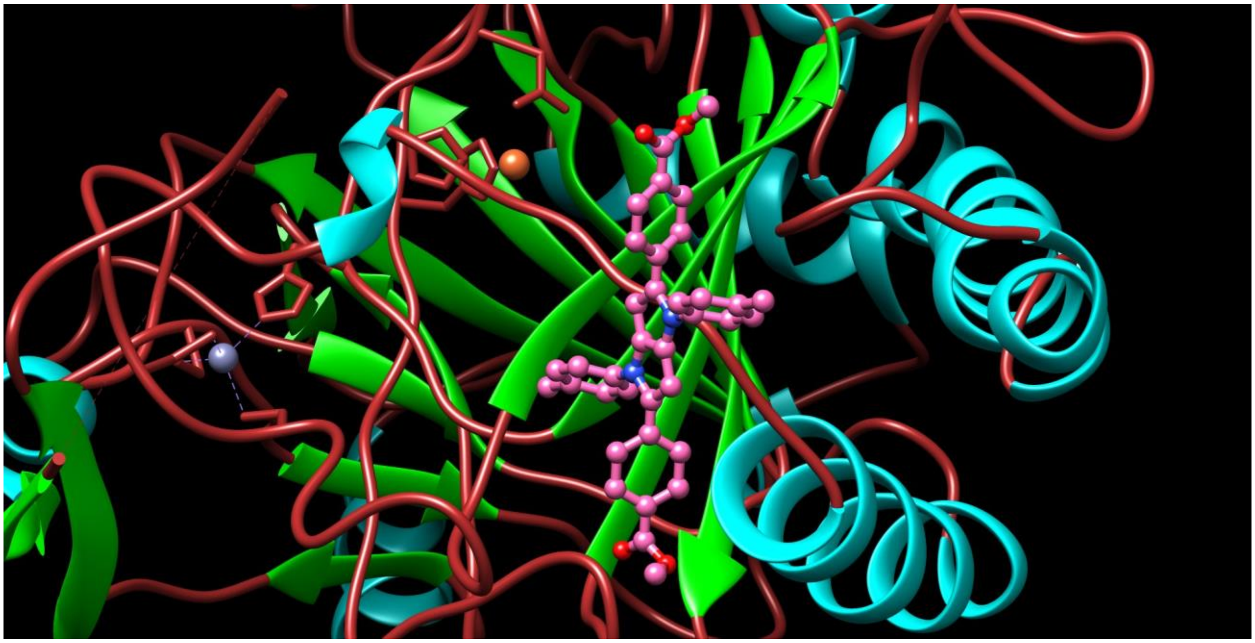

Figure S20. Close up view of the TET1 active site showing docked compound **7** in a sphere representation. The Fe(II) ion is displayed as a crossed orange sphere, the protein residues involved in contacts with compound **7** (pink sticks) are shown as sticks. The hydrogen bonds and carbon hydrogen bond are indicated by purple and yellow dotted lines, respectively.

Inhibition of TET1 protein

Table **S1.** Residual TET1 protein activity on the concentration of compound **1.**

Log c (751/compound 1)	average residual TET activity (%)
4	5.107745
5	19.97357
6	56.08355
7	82.58792

Toxicity (MTT assay)

Caov-3

Table **S2.** Viability of Caov-3 cells in the presence of compound **1**.

Cell viability %			
**20 uM**	28.51596	35.73407	39.69061
**15 uM**	35.10045	41.90383	45.92074
**10 uM**	45.06162	53.56232	43.67450
**7.5 uM**	56.44099	51.67812	65.52312
**5 uM**	50.93702	48.77331	58.08483
**2.5 uM**	55.86696	71.78777	55.75334
**1 uM**	81.16343	71.06968	89.65883
**750 nM**	78.45686	95.40726	77.66887
**500 nM**	91.35573	100.9421	76.34775
**100 nM**	97.8727	80.60844	94.80077
**Control**	100	100	100

Table **S3.** Viability of Caov-3 cells in the presence of compound **2**.

Cell viability %			
**20 uM**	28.42292	25.29919	23.24628
**15 uM**	42.75603	50.9752	42.32679
**10 uM**	52.13448	51.36046	52.94626
**7.5 uM**	73.28024	79.19576	67.96892
**5 uM**	59.21257	86.03419	70.4727
**2.5 uM**	64.6096	82.47049	61.81465
**1 uM**	78.50034	67.15627	48.54306
**750 nM**	73.36872	52.90151	76.28723
**500 nM**	76.19996	78.8105	77.84582
**100 nM**	95.32425	105.779	93.87006
**Control**	100	100	100

Table **S4.** Viability of Caov-3 cells in the presence of compound **3**.

**Cell viability %**			
**20 uM**	35.49563	34.97841	37.90087
**15 uM**	58.99942	57.50729	62.97376
**10 uM**	65.43991	67.36582	81.12244
**7.5 uM**	83.03623	61.93708	104.0087
**5 uM**	70.6153	67.61258	86.51603
**2.5 uM**	85.27889	89.38926	98.68805
**1 uM**	89.3617	86.92165	97.81341
**750 nM**	86.6015	81.49291	112.9009
**500 nM**	93.50201	75.38556	98.76093
**100 nM**	84.41633	80.39359	71.13703
**Control**	100	100	100

Table **S5.** Viability of Caov-3 cells in the presence of compound **4**.

**Cell viability %**			
**20 uM**	14.32384	14.90858	14.49109
**15 uM**	19.49414	17.58087	17.59632
**10 uM**	42.88256	41.06892	42.6107
**7.5 uM**	53.11536	53.65682	54.16907
**5 uM**	50.70943	80.94234	55.26165
**2.5 uM**	74.64528	110.1266	69.69523
**1 uM**	100.802	98.73418	92.86946
**750 nM**	92.84392	102.8833	94.07706
**500 nM**	104.1332	102.391	108.5681
**100 nM**	68.5943	84.94756	82.17366
**Control**	100	100	100

Table **S6.** Viability of Caov-3 cells in the presence of compound **5**.

**Cell viability %**			
**20 uM**	17.9126	16.1941	14.82686
**15 uM**	31.48711	34.22252	36.44149
**10 uM**	29.50216	41.31352	47.32635
**7.5 uM**	62.47383	49.34999	63.65028
**5 uM**	83.63463	81.63093	70.92022
**2.5 uM**	106.339	69.33987	79.185
**1 uM**	115.5595	95.47527	128.3145
**750 nM**	97.71646	117.0859	120.9681
**500 nM**	90.56137	116.6807	137.4211
**100 nM**	124.7159	131.0561	82.39908
**Control**	100	100	100

Table **S7.** Viability of Caov-3 cells in the presence of compound **6**.

Cell viability %			
**20 uM**	17.40035	17.33918	16.87916
**15 uM**	36.28942	42.46159	49.26344
**10 uM**	52.61726	73.89153	61.73714
**7.5 uM**	57.99583	65.62553	91.09419
**5 uM**	62.99023	65.69306	100.7653
**2.5 uM**	67.85657	93.922	75.10942
**1 uM**	64.71906	111.7508	115.9087
**750 nM**	66.96014	65.96319	78.49627
**500 nM**	89.11477	125.1899	99.08169
**100 nM**	90.2569	126.2705	118.7488
**Control**	100	100	100

H-1299

Table **S8.** Viability of H-1299 cells in the presence of compound **1**.

Cell viability %			
**20 uM**	19.24406	17.01043	14.67647
**15 uM**	23.87986	23.04042	23.96276
**10 uM**	35.21357	36.64265	31.8986
**7.5 uM**	38.35093	36.84003	36.66628
**5 uM**	57.68489	61.73559	69.22959
**2.5 uM**	68.0251	76.07293	72.38632
**1 uM**	69.85522	81.23464	63.29924
**750 nM**	97.30258	84.13106	122.0664
**500 nM**	114.5756	80.35216	108.7959
**100 nM**	86.71852	74.42021	77.19802
**Control**	100	100	100

Table **S9.** Viability of H-1299 cells in the presence of compound **2**.

Cell viability %			
**20 uM**	28.59897	32.03724	28.81821
**15 uM**	39.77581	39.64275	37.79295
**10 uM**	45.51456	38.48482	47.34705
**7.5 uM**	42.29877	36.16895	50.74895
**5 uM**	53.16155	53.26162	57.69069
**2.5 uM**	73.18867	66.9068	60.81677
**1 uM**	72.40433	69.86743	82.46944
**750 nM**	74.14295	70.73588	81.48872
**500 nM**	71.47733	66.22258	72.35567
**100 nM**	77.6381	61.36715	71.29831
**Control**	100	100	100

Table **S10.** Viability of H-1299 cells in the presence of compound **3**.

**Cell viability %**			
**20 uM**	31.42255	35.73111	34.4596
**15 uM**	58.89207	44.57329	46.49157
**10 uM**	47.85276	61.93151	65.75698
**7.5 uM**	56.61426	68.90741	84.49209
**5 uM**	85.7362	93.25965	99.67511
**2.5 uM**	123.9575	110.6978	112.6272
**1 uM**	70.17579	93.78511	104.6587
**750 nM**	90.73998	102.5498	126.8789
**500 nM**	146.8316	115.9449	119.3443
**100 nM**	132.2567	108.7232	102.9023
**Control**	100	100	100

Table **S11.** Viability of H-1299 cells in the presence of compound **4**.

**Cell viability %**			
**20 uM**	7.35088	7.889723	6.314614
**15 uM**	11.0926	10.45612	11.77551
**10 uM**	14.81916	21.63938	18.83578
**7.5 uM**	35.84548	38.28207	33.07157
**5 uM**	66.67298	68.73663	52.75386
**2.5 uM**	77.6406	77.91344	68.92042
**1 uM**	88.01973	73.99385	66.83116
**750 nM**	86.06311	86.19959	81.88825
**500 nM**	110.8313	69.07882	86.71517
**100 nM**	109.7831	109.0835	92.98296
**Control**	100	100	100

Table **S12.** Viability of H-1299 cells in the presence of compound **5**.

**Cell viability %**			
**20 uM**	31.42255	35.73111	28.72878
**15 uM**	46.49157	44.57329	49.80137
**10 uM**	47.85276	61.93151	54.82123
**7.5 uM**	56.61426	68.90741	70.44059
**5 uM**	85.7362	93.25965	83.0986
**2.5 uM**	105.6814	110.6978	93.89671
**1 uM**	59.82921	93.78511	88.11845
**750 nM**	77.36145	98.71354	102.5498
**500 nM**	125.183	115.9449	119.3443
**100 nM**	112.7571	101.9931	108.7232
**Control**	100	100	100

Table **S13.** Viability of H-1299 cells in the presence of compound **6**.

Cell viability %			
**20 uM**	34.39408	33.42316	31.59845
**15 uM**	38.6779	36.41601	29.23822
**10 uM**	54.81208	52.11373	46.78871
**7.5 uM**	63.73957	53.98429	50.35933
**5 uM**	69.39882	77.63368	53.79378
**2.5 uM**	77.44904	63.25052	56.68356
**1 uM**	86.37654	69.89151	61.19579
**750 nM**	90.21244	83.61392	74.74846
**500 nM**	82.0418	69.87655	74.97541
**100 nM**	74.56842	77.16424	73.26575
**Control**	100	100	100

BT-20

Table **S14.** Viability of BT-20 cells in the presence of compound **1**.

Cell viability %			
**20 uM**	19.84508	18.85411	15.23017
**15 uM**	33.46688	32.99136	30.01959
**10 uM**	41.4797	44.0939	37.78159
**7.5 uM**	41.17171	50.1262	42.238
**5 uM**	56.78419	56.30994	56.39079
**2.5 uM**	69.15064	55.65371	54.6523
**1 uM**	102.5641	98.92008	100.049
**750 nM**	91.1859	98.59611	95.51909
**500 nM**	87.76709	106.1555	91.84623
**100 nM**	101.7094	100.0252	99.53477
**Control**	100	100	100

Table **S15.** Viability of BT-20 cells in the presence of compound **2**.

Cell viability %			
**20 uM**	28.29374	24.60878	32.37022
**15 uM**	46.67927	49.89904	44.53966
**10 uM**	45.0324	57.49622	51.12635
**7.5 uM**	60.23218	62.61989	57.00293
**5 uM**	73.8391	79.32862	68.97649
**2.5 uM**	98.61111	100.513	96.3761
**1 uM**	123.7041	111.0298	97.08619
**750 nM**	88.88888	105.3004	102.8159
**500 nM**	89.79482	89.90409	93.14397
**100 nM**	89.52991	96.38229	111.3369
**Control**	100	100	100

Table **S16.** Viability of BT-20 cells in the presence of compound **3**.

Cell viability %			
**20 uM**	40.96308	33.39072	29.51477
**15 uM**	48.94036	53.93698	48.1349
**10 uM**	62.90925	60.85546	57.18601
**7.5 uM**	73.13243	71.92611	72.15611
**5 uM**	85.1299	91.78971	101.4304
**2.5 uM**	99.35225	99.33788	98.89039
**1 uM**	103.802	95.2857	94.69757
**750 nM**	99.71837	90.49196	101.3318
**500 nM**	95.26861	90.62438	106.8284
**100 nM**	83.41196	73.85949	70.55268
**Control**	100	100	100

Table **S17.** Viability of BT-20 cells in the presence of compound **4**.

Cell viability %			
**20 uM**	8.369255	9.863568	12.91981
**15 uM**	8.055758	8.453554	10.83292
**10 uM**	20.10301	18.92421	21.27463
**7.5 uM**	37.492	44.30446	44.18081
**5 uM**	73.46471	77.51811	61.61889
**2.5 uM**	82.73526	106.2024	110.5893
**1 uM**	96.26043	100.5483	88.50558
**750 nM**	103.1798	88.69378	109.7163
**500 nM**	77.83127	90.8088	82.56707
**100 nM**	89.04617	76.96978	98.11308
**Control**	100	100	100

Table **S18.** Viability of BT-20 cells in the presence of compound **5**.

Cell viability %			
**20 uM**	20.91107	18.3694	26.24032
**15 uM**	28.47104	28.44734	34.27277
**10 uM**	41.28907	52.47014	37.54404
**7.5 uM**	45.69905	52.633	55.7373
**5 uM**	60.98861	87.48642	89.10418
**2.5 uM**	80.30046	100.57	104.0262
**1 uM**	98.95808	92.10098	100.302
**750 nM**	86.63815	80.99891	102.8391
**500 nM**	97.43155	90.47231	107.0261
**100 nM**	85.99467	94.73398	89.5899
**Control**	100	100	100

Table **S19.** Viability of BT-20 cells in the presence of compound **6**.

Cell viability %			
**20 uM**	18.802	24.04454	23.03115
**15 uM**	37.77038	26.72284	42.10711
**10 uM**	36.17304	29.6118	40.91705
**7.5 uM**	50.48253	49.86458	58.52293
**5 uM**	85.79034	70.29792	79.41897
**2.5 uM**	89.15141	84.17092	92.78626
**1 uM**	91.21464	79.29582	90.75954
**750 nM**	85.05824	84.44176	98.05343
**500 nM**	79.86689	79.34897	86.48855
**100 nM**	103.6273	95.0647	99.23664
**Control**	100	100	100

U-118 MG

Table **S20.** Viability of **U-118 MG** cells in the presence of compound **1**.

Cell viability %			
**20 uM**	32.43007	38.37077	42.77049
**15 uM**	52.86713	49.75257	52.18157
**10 uM**	49.80769	65.4465	62.16216
**7.5 uM**	65.89161	73.35363	73.21463
**5 uM**	64.50323	72.2848	61.20133
**2.5 uM**	59.82518	71.64119	61.0448
**1 uM**	61.64336	65.24553	66.50362
**750 nM**	75.25695	75.76429	76.03209
**500 nM**	81.23335	77.57442	78.28213
**100 nM**	74.91259	89.41759	92.95637
**Control**	100	100	100

Table **S21.** Viability of **U-118 MG** cells in the presence of compound **2**.

Cell viability %			
**20 uM**	39.14967	44.6621	48.65798
**15 uM**	51.07196	55.67384	56.44768
**10 uM**	57.92607	65.41163	61.06994
**7.5 uM**	66.32476	62.32276	65.79428
**5 uM**	62.14151	65.49889	62.22748
**2.5 uM**	70.66434	64.2773	70.34197
**1 uM**	84.08913	82.07757	80.29337
**750 nM**	69.99316	77.01671	67.38152
**500 nM**	63.95961	68.29109	66.75732
**100 nM**	82.47858	87.05401	87.05401
**Control**	100	100	100

Table **S22.** Viability of **U-118 MG** cells in the presence of compound **3**.

Cell viability %			
**20 uM**	35.11642	30.46595	32.81925
**15 uM**	42.59714	46.07461	47.08923
**10 uM**	45.5567	53.28832	53.00398
**7.5 uM**	48.34217	52.95491	55.16316
**5 uM**	57.14173	68.44211	68.47725
**2.5 uM**	64.67516	61.47371	71.57868
**1 uM**	75.91201	71.03783	66.44352
**750 nM**	70.23028	72.44006	70.84993
**500 nM**	87.97183	59.47373	56.29184
**100 nM**	90.89415	99.64991	109.711
**Control**	100	100	100

Table **S23.** Viability of **U-118 MG** cells in the presence of compound **4**.

Cell viability %			
**20 uM**	15.17047	15.6756	12.30857
**15 uM**	21.16659	21.17125	19.81549
**10 uM**	29.97722	29.03249	31.25504
**7.5 uM**	24.82409	24.18593	24.16683
**5 uM**	73.68801	72.70924	76.45973
**2.5 uM**	78.81056	87.60953	83.16356
**1 uM**	88.82355	94.85053	87.09975
**750 nM**	88.64679	93.98062	97.44762
**500 nM**	81.50854	94.34568	91.80472
**100 nM**	89.69409	88.01342	106.1964
**Control**	100	100	100

Table **S24.** Viability of **U-118 MG** cells in the presence of compound **5**.

Cell viability %			
**20 uM**	21.02625	24.2419	18.84402
**15 uM**	28.8956	30.29581	23.7185
**10 uM**	45.49669	44.7379	34.30884
**7.5 uM**	47.16612	44.60394	36.57689
**5 uM**	65.29145	61.9341	54.54753
**2.5 uM**	58.217	62.66636	72.48403
**1 uM**	87.35514	91.36398	94.34816
**750 nM**	101.0241	97.48939	72.15236
**500 nM**	91.72102	83.56483	99.83889
**100 nM**	97.80089	97.50102	97.94004
**Control**	100	100	100

Table **S25.** Viability of **U-118 MG** cells in the presence of compound **6**.

**Cell viability %**			
**20 uM**	22.54731	18.63575	22.51363
**15 uM**	28.04868	29.05686	32.77937
**10 uM**	49.60407	51.17363	52.18558
**7.5 uM**	45.91981	45.39844	56.877
**5 uM**	59.03976	63.22346	58.23376
**2.5 uM**	63.02409	69.74832	76.68138
**1 uM**	68.44211	80.40844	81.53043
**750 nM**	67.1918	73.07008	81.92678
**500 nM**	73.47671	83.7778	86.54896
**100 nM**	98.13287	91.37361	92.91959
**Control**	100	100	100

HF-P4

Table **S26.** Viability of **HF-P4** cells in the presence of compound **1**.

**Cell viability %**			
**20 uM**	63.95658	64.64763	61.04274
**15 uM**	67.05804	59.85663	63.15062
**10 uM**	70.53611	75.93788	65.94317
**7.5 uM**	77.31501	87.02509	78.04878
**5 uM**	80.70447	90.58038	81.47236
**2.5 uM**	94.32875	106.4486	88.2524
**1 uM**	105.1617	114.0028	114.925
**750 nM**	93.95214	108.0378	98.14276
**500 nM**	106.2251	109.6499	91.29559
**100 nM**	101.152	110.5137	108.5444
**Control**	100	100	100

Table **S27.** Viability of **HF-P4** cells in the presence of compound **2**.

**Cell viability %**			
**20 uM**	60.27913	51.8655	56.81361
**15 uM**	69.42844	82.12665	69.57623
**10 uM**	83.07232	82.91517	77.13135
**7.5 uM**	84.40811	87.78973	98.14276
**5 uM**	103.5928	91.30227	88.76707
**2.5 uM**	91.9392	112.4492	91.40747
**1 uM**	107.1396	107.7419	92.48154
**750 nM**	100.1551	106.3799	108.6149
**500 nM**	120.4253	121.1654	117.1851
**100 nM**	112.667	100.0956	124.6811
**Control**	100	100	100

Table **S28.** Viability of **HF-P4** cells in the presence of compound **3**.

**Cell viability %**			
**20 uM**	59.02883	62.77504	65.696
**15 uM**	65.59934	66.39747	65.2
**10 uM**	82.57799	74.36797	72.544
**7.5 uM**	78.68825	83.18117	77.088
**5 uM**	85.84565	90.27387	73.392
**2.5 uM**	87.06795	92.78441	93.77668
**1 uM**	100.1686	97.12644	98.88
**750 nM**	106.525	98.1566	91.216
**500 nM**	99.88198	100.1756	103.312
**100 nM**	95.80172	96.12008	98.08001
**Control**	100	100	100

Table **S29.** Viability of **HF-P4** cells in the presence of compound **4**.

**Cell viability %**			
**20 uM**	36.4007	38.67986	44.62788
**15 uM**	45.12022	53.93521	54.86213
**10 uM**	59.88019	62.49443	65.68416
**7.5 uM**	66.93568	70.27355	58.23608
**5 uM**	74.85648	73.33582	61.31848
**2.5 uM**	74.54031	86.63056	76.23162
**1 uM**	85.95557	81.66592	86.70585
**750 nM**	84.8196	85.09943	92.37617
**500 nM**	94.85814	87.47438	94.86558
**100 nM**	102.3796	100.6236	114.7117
**Control**	100	100	100

Table **S30.** Viability of **HF-P4** cells in the presence of compound **5**.

**Cell viability %**			
**20 uM**	52.22591	55.03193	53.23501
**15 uM**	62.99003	60.11892	45.66372
**10 uM**	76.65559	58.3808	56.93216
**7.5 uM**	74.4629	71.79035	64.34612
**5 uM**	83.96829	65.29699	72.64504
**2.5 uM**	96.80687	91.7616	90.46214
**1 uM**	105.1971	86.79821	100.59
**750 nM**	84.18605	95.78926	102.0649
**500 nM**	105.4928	98.20993	101.0423
**100 nM**	104.2968	105.3072	105.0738
**Control**	100	100	100

Table **S31.** Viability of **HF-P4** cells in the presence of compound **6**.

**Cell viability %**			
**20 uM**	48.01772	42.61595	43.53982
**15 uM**	62.45847	54.90236	58.15143
**10 uM**	66.11296	61.41172	64.7001
**7.5 uM**	62.32558	61.49309	69.34596
**5 uM**	84.31894	73.0472	65.60473
**2.5 uM**	94.52935	75.57806	74.70994
**1 uM**	84.38669	84.11311	85.78171
**750 nM**	97.81986	92.16844	90.67847
**500 nM**	102.5471	95.78926	101.8289
**100 nM**	114.4851	90.23597	95.39749
**Control**	100	100	100
